# Protective Effect of Uric Acid on ox-LDL-Induced HUVECs Injury via Keap1-Nrf2-ARE Pathway

**DOI:** 10.1155/2021/5151168

**Published:** 2021-11-01

**Authors:** Yajuan Lin, Yunpeng Xie, Zhujing Hao, Hailian Bi, Yang Liu, Xiaolei Yang, Yunlong Xia

**Affiliations:** Department of Cardiology, First Affiliated Hospital of Dalian Medical University, Dalian, Liaoning, China

## Abstract

Uric acid is an effective antioxidant. Oxidized low-density lipoprotein (ox-LDL) is derived from circulating LDL and promotes atherosclerosis. The Keap1-Nrf2-ARE pathway is a key body pathway involved in protection against internal and external oxidative damages. The role of uric acid on vascular endothelial function damaged by ox-LDL, and its effect on the Keap1-Nrf2-ARE pathway has not been fully explored. HUVECs were treated with different concentrations of uric acid and ox-LDL to explore the effect of uric acid in vitro. Cell phenotype was determined by cytometry and Western blot. Nuclear translocation of Nrf2 was determined by immunofluorescence. Coimmunoprecipitation was used to determine the level of Nrf2 ubiquitination. A microfluidic device was used to mimic the vascular environment in the body, and the level of mRNA levels of inflammatory factors was determined by RT-PCR. The findings of this study show that suitable uric acid can significantly reduce endothelial damage caused by ox-LDL, such as oxidative stress, inflammation, and increased adhesion. In addition, uric acid reduced Nrf2 ubiquitination and increased nuclear translocation of Nrf2 protein, thus activating the Keap1-Nrf2-ARE pathway and playing a protective role. Interestingly, the effects of UA were significantly inhibited by administration of Brusatol, an inhibitor of Nrf2. In summary, suitable concentrations of uric acid can alleviate the oxidative stress level of endothelial cells through Nrf2 nuclear translocation and further protect cells from damage.

## 1. Introduction

Oxidized low-density lipoprotein (ox-LDL) promotes atherosclerosis (AS). Oxidized LDL level increases during the occurrence of AS, which can lead to damage vascular endothelial cells [1]. Several studies report that vascular endothelial cell damage and functional changes are initial manifestations of the occurrence and development of AS [2]. ox-LDL deposited in the vascular wall induces vascular endothelial cell apoptosis. ox-LDL-induced oxidative stress is a major cause of endothelial cell injury [3]. ox-LDL causes apoptosis of endothelial cells by inducing intracellular oxidative stress and endoplasmic reticulum stress [2, 4]. Therefore, inhibiting ox-LDL-mediated endothelial injury is a potential strategy for preventing or slowing progression of AS.

Uric acid (UA) is the final metabolite of purine catabolism in humans. Studies report that hyperuricemia can induce endothelial dysfunction and lead to the occurrence and development of a variety of cardiovascular diseases [5–11]. However, in an in vitro study, it was demonstrated that the antioxidant effect of UA is equal to that of ascorbate, a significant antioxidant in plasma [12]. Human plasma urate levels are significantly greater than ascorbate levels. As a result, UA is estimated to be responsible for neutralizing more than half of the free radicals in human blood [13]. UA effectively removes reactive oxygen free radicals, hydroxyl free radicals, and peroxides in the body. In addition, it blocks the nitrification reaction and chelates metal ions such as iron ions, thus reducing oxidative stress reaction in the body and maintaining immune defense ability of the body [14–16]. A previous study reports that the appropriate concentration of UA (300 *μ*M) can significantly increase the activity of neurons and reduce the production of reactive oxygen species [17]. In addition, significantly low levels of uric acid are associated with Alzheimer's disease, cardiovascular disease, diabetes, and other neurological diseases. Therefore, we hypothesized that appropriate uric acid concentration can be effectively alleviated of ox-LDL-mediated endothelial injury.

The Kelch-like ECH-associated protein 1-nuclear factor erythroid 2-related factor 2-antioxidant response elements (Keap1-Nrf2-ARE) pathway is one of the most important defense mechanisms against oxidative stress [18] and is associated with a number of oxidative stress-related diseases, including cancer, neurodegenerative diseases, cardiovascular diseases, and aging [19]. Nrf2 signaling pathway activation can modulate expression of genes implicated in detoxification and antioxidant defense functions, such as NAD (P) H: quinone oxidoreductase 1, superoxide dismutase, heme oxygenase-1 (HO-1) and catalase, thioredoxin reductase [20]. In the physiological environment, Nrf2 is located in the cytoplasm and it binds to Keap1 which controls Nrf2 activity. Oxidative or electrophilic stress induces a conformational change of Keap1 or directly promotes phosphorylation of Nrf2. Therefore, Nrf2 is segregated from Keap1 and translocated to the nucleus to effectively combine with antioxidant reaction components (ARE). As a result, it upregulates transcription of antioxidant and detoxifying genes [21]. Studies report that Nrf2 activation can protect endothelial cells from oxidative damage [22] and inflammatory response [23].

In this study, it was hypothesized that suitable concentrations of UA can minimize endothelial cell damage caused by ox-LDL. Therefore, the mechanism of action of uric acid in alleviating ox-LDL-induced damage and its effect on the Nrf2 pathway in human umbilical vein endothelial cells (HUVECs) were explored.

## 2. Materials and Methods

### 2.1. Materials

UA was obtained from Sigma-Aldrich (St. Louis, MO, USA). ox-LDL (UBC-ox-LDL5) was obtained from Yiyuanbiotech (Guangzhou, China) with a 2.1-2.5 mg/mL concentration. Brusatol (BT, a Nrf2 inhibitor) was purchased from MCE (Burlington, NJ, USA) and was dissolved in dimethyl sulfoxide (DMSO, the final concentration < 0.1%). An antibody against ICAM-1 was obtained from Cell Signaling Technology (Danvers, MA, USA). Dihydroethidine (DHE) was obtained from Sigma-Aldrich (St. Louis, MO, USA). Endothelial Cell Medium (ECM) was obtained from ScienCell (San Diego, CA, USA). Antibodies against histone H3 (17168-1-AP), HO-1 (10701-1-AP), NQO1 (11451-1-AP), and *α*-tubulin (66031-1-Ig) were purchased from Proteintech (Wuhan, Hubei, China). Antibodies against Keap1 (ab139729) and Nrf2 (ab137550) were purchased from Abcam (Cambridge, MA, USA).

### 2.2. Cell Culture

HUVECs and THP-1 cell were donated by Yang et al. [24]. HUVECs were incubated in ECM contained with 5% fetal bovine serum (FBS), containing 1% penicillin/streptomycin (P/S) and 1% endothelial cell growth factor at 5% CO_2_ and 37°C. THP-1 cells were incubated in RPMI1640 containing 10% FBS and 1% P/S. After achieving ~70% confluence, cells were divided into the five groups. The groups included a control group, in which cells were grown in ECM; an UA group, in which cells were grown for 24 h in different concentrations of UA (1, 2, 3, 5, 6, 9, 12, 15, and 18 mg/dL); an ox-LDL group, in which cells were grown for 24 h in different concentrations of ox-LDL (10, 20, 50, 100, and 200 *μ*g/mL); an ox-LDL+UA group, in which cells were grown in different concentrations of UA and ox-LDL (100 *μ*g/mL) for 24 h; and an ox-LDL+UA+brusatol group, in which cells were pretreated with brusatol (300 nM) for 2 h before incubation with UA (5 mg/dL)+ox-LDL (100 *μ*g/mL) for 24 h.

### 2.3. Cell Viability Assay

Cell Counting Kit-8 (CCK-8) (KeyGEN Biotech, Jiangsu, Nanjing, China) was used to explore cell viability of HUVECs following the manufacturer's instructions. In summary, HUVECs were incubated in a 96-well plate and were treated with CCK-8 diluted in culture medium (1 : 10) for 4 hours. Cell viability, using a microplate reader (M1000 PRO, Tecan, USA), was then determined at 450 nm. A total of 5 replicates were used.

### 2.4. MDA, ET-1, and NO Levels

MDA level was measured using a Nanjing Jiancheng assay kit to determine lipid peroxidation. Level of MDA was measured using the assay kit following the manufacturer's instructions. Human plasma endothelin-1 level (ET-1) was determined using CUSABIO BIOTECH human ET-1 enzyme-linked immunosorbent assay (ELISA) kit (Wuhan, China). The NO level was determined by the Classic Griess Reagent method using a NO production assay kit.

### 2.5. Nrf2 Nuclear Translocation

HUVECs were fixed with 4% paraformaldehyde for 15 minutes and blocked with 10% goat serum for 60 minutes and with 2‰ Triton X-100 for 15 minutes at room temperature. Cells were then grown overnight at 4°C with anti-Nrf2 rabbit polyclonal antibody (1 : 500 dilution) and then probed for 1 h with goat anti-rabbit secondary antibody Alexa Fluor ® 488 (1 : 500) at room temperature under dark conditions. Nuclear staining was performed using DAPI (100 ng/mL) for 5 minutes and then observed under a microscope (Nikon).

### 2.6. Western Blot Analysis

HUVECs were added to ice-cold RIPA buffer containing inhibitors and PMSF (100 mM; Solarbio, Beijing, CHN) and sonicated. The BCA method was used to determine protein concentration. Proteins were transferred to SDS-PAGE gel and separated by electrophoresis; then, they were blotted onto the PVDF membrane (Millipore, Billerica, MA, USA). Nrf2 (1 : 500), NQO1 (1 : 1000), Keap1 (1 : 500), HO-1 (1 : 1000), *α*-Tubulin (1 : 2500), and Histone-3 (1: 500) antibodies were used. The blots were generated using enhanced chemiluminescent system (ECL Plus, Thermo Fisher, Waltham, MA, USA), and FluorChem M system (ProteinSimple, San Jose, CA, USA) was used for signal acquisition. ImageJ software was used for quantitative analysis of protein, which were then standardized using the concentration of endogenous *α*-tubulin and histone-3.

### 2.7. Realtime PCR

Total RNA was extracted from HUVECs using TRIzol reagent and reverse transcribed using a reverse transcription kit to synthesize first-strand cDNA (RR047A; Takara, Tokyo, JPN). qPCR amplification was conducted using SYBR Green on an Applied Bio Systems 7500 Real-Time PCR system (Applied Bio systems/Thermos Fisher Scientific, Foster City, CA, USA). cDNA was amplified using a primer pair specific to human TGF*β* IL-1*β*, IL-6, NOX 4, TNF*α*, and GADPH. Relative levels of mRNA were standardized to endogenous control (GAPDH) levels. Primer sequences are presented in Table 1.

### 2.8. Immunofluorescence Staining

HUVECs were fixed on coverslips for 15 minutes at room temperature with 4% paraformaldehyde. After blocking cells in 10% goat serum for 1 h and 1‰ Triton-X 100 for 15 minutes, ICAM-1 antibody diluted at 1 : 100 was added and the mixture was incubated overnight at 4°C. After washing three times with PBS, the second Alexa Fluor ® 555 antibody at a concentration of 1 *μ*g/mL was added and the mixture incubated for 1 h at room temperature. Nuclei were stained with 100 ng/mL DAPI for 5 min. 1 : 200 dilutions of dihydroethidine (DHE) were added for 1 hour at room temperature. Covers were then sealed, and cells were observed under fluorescence microscopy with an antifade mounting medium (magnification, ×20).

### 2.9. Immunoprecipitation

Immunoprecipitation was used to determine the level of Nrf2 ubiquitination as described previously [25]. In summary, cell extracts were treated with a primary antibody (4 *μ*g) and incubated overnight at 4°C. Cells were then centrifuged at 3000 rpm at 4°C. Bound proteins were eluted in 4x sample buffer by boiling beads. Precipitated proteins were separated by SDS-PAGE using 6 percent gels followed by Western blot analysis. ImageJ was used for analysis and quantification of immunoblot data.

### 2.10. Cell Adhesion Assay

Cells were added into 6-well plates, and the adhesion assay was performed to determine the effect of uric acid and ox-LDL on THP-1 cell adhesion to HUVECs. HUVECs stimulated with UA or ox-LDL were dyed with green fluorescence (Mito-Tracker Green, Solarbio, Beijing, China) and incubated on the lower Transwell chamber (8.0 *μ*m diameter pore, Corning) at 5% CO_2_ at 37°C for 24 h. After 24 h in the upper chamber DiI (red fluorescence), prestained THP-1 cells were cocultured with HUVECs for 4 h. After washing, the adhesion rate of THP-1 cells was determined by observation under a fluorescence microscope (Olympus BX50).

### 2.11. Development of Microfluidic Devices

As previously described [26], microdevices were designed using standard microfabrication techniques. PDMS prepolymer (10 : 1 = base: curing agent) was degassed and filled in equipped masters before sealing it irreversibly with a clean glass substrate. The unit used for cell culture had one entry, one outlet, and four chambers measuring 200 *μ*m in height, 1 mm in width, and 2 cm in length.

### 2.12. Statistical Analysis

The GraphPad Prism-7.0 application was used to do all statistical calculations. The mean ± standard deviation was used to express all of the data. To identify differences among various groups, one-way or two-way ANOVA was used, followed by a Tukey post hoc test for pairwise comparison. *P* < 0.05 was used to determine statistical differences.

## 3. Results and Discussion

### 3.1. Effect of Uric Acid and ox-LDL on HUVECs

**Figure 1:**
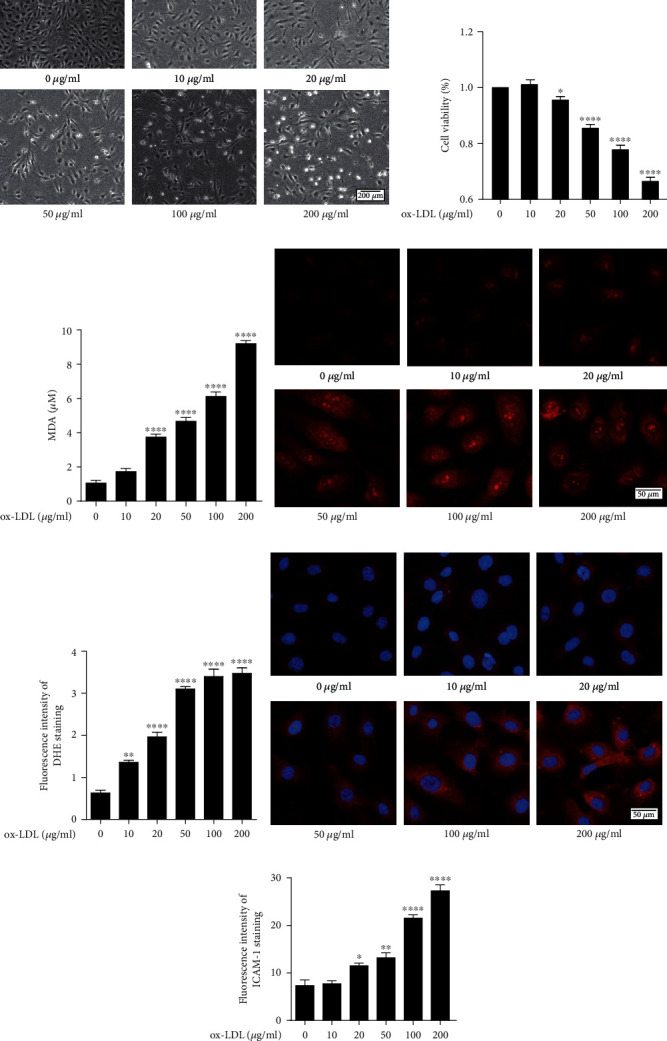
HUVEC injury caused by ox-LDL stimulation at different concentrations HUVECs were stimulated with 0, 10, 20, 50, 100, and 200 *μ*g/mL ox-LDL for 24 h. Changes in (a) cell morphology in different groups. (b) Cell survival rate in different groups by CCK-8 assay. Scale bar = 200 *μ*m. (c) Changes in MDA levels. (d, e) DHE immunofluorescence image and densitometry analysis of immunofluorescent intensity of DHE. Scale bar = 50 *μ*m. (f, g) ICAM1 immunofluorescence image and densitometry analysis of immunofluorescent intensity of ICAM1. Scale bar = 50 *μ*m. DHE: dihydroethidium; HUVECs: human umbilical vein endothelial cells; ox-LDL: oxidized low-density lipoprotein; MDA: malondialdehyde. The statistical analyses were done using GraphPad Prism V-7.0 software. Data are expressed as the mean ± standard deviation (*n* = 3). One-way ANOVA was used in statistical analyses (*n* = 3/group). ^∗^*P* < 0.05, ^∗∗^*P* < 0.01, ^∗∗∗^*P* < 0.001, and ^∗∗∗∗^*P* < 0.0001 versus the control.

The CCK-8 assay was used to investigate the effect of varying ox-LDL concentrations on HUVEC viability. Cell viability gradually decreased with an increase in ox-LDL concentration (Figures 1(a) and 1(b)). MDA and DHE staining were used for determination of ROS levels in HUVECs after treatment different concentrations of ox-LDL. Increased ox-LDL concentration resulted in a considerable increase in MDA levels (Figure 1(c)). The intensity of DHE staining increased as the concentration of ox-LDL increased, according to the findings (Figures 1(d) and 1(e)). ICAM-1 staining was used to detect occurrence of inflammatory response after treatment with different concentrations of ox-LDL. Similarly, as the concentration of ox-LDL increased, the fluorescence intensity of ICAM-1 increased considerably (Figures 1(f)–1(g)). Treatment with different high concentrations of uric acid (6 to 18 mg/dL) showed strong (*P* < 0.05) cytotoxicity towards HUVECs.

**Figure 2:**
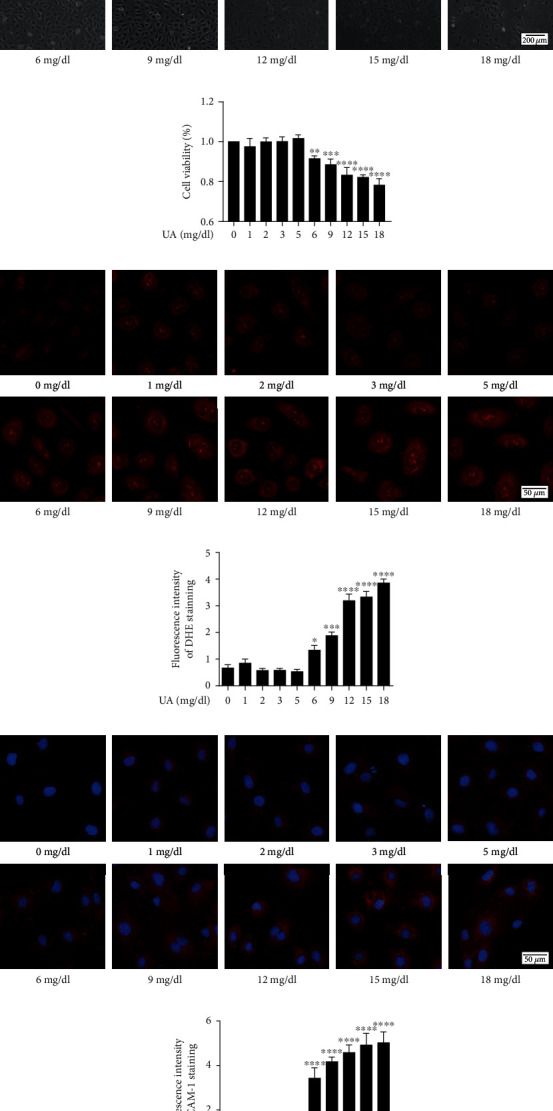
Changes of HUVECs under the stimulation of different concentrations of uric acid HUVECs were stimulated with 0, 1, 2, 3, 5, 6, 9, 12, 15, and 18 mg/dL uric acid for 24 h. Changes in (a) cell morphology in different groups. Scale bar = 200 *μ*m. (b) Cell survival rate in different groups by CCK-8 assay. (c, d) DHE immunofluorescence image and densitometry analysis of immunofluorescent intensity of DHE. Scale bar = 50 *μ*m. (e, f) ICAM1 immunofluorescence image and densitometry analysis of immunofluorescent intensity of ICAM1. Scale bar = 50 *μ*m. DHE: dihydroethidium; HUVECs: human umbilical vein endothelial cells. The statistical analyses were done using GraphPad Prism V-7.0 software. Data are expressed as the mean ± standard deviation (*n* = 3). One-way ANOVA was used in statistical analyses (*n* = 3/group). ^∗^*P* < 0.05, ^∗∗^*P* < 0.01, ^∗∗∗^*P* < 0.001, and ^∗∗∗∗^*P* < 0.0001 versus the control.

Treatment with concentration of UA ≥ 6 mg/dL significantly decreased cell viability (Figures 2(a) and 2(b)). In addition, a significant increase in fluorescence intensity of DHE and ICAM-1 was observed (Figures 2(c)–2(f)). However, treatment with 1-5 mg/dL uric acid had no effect on cell viability.

**Figure 3:**
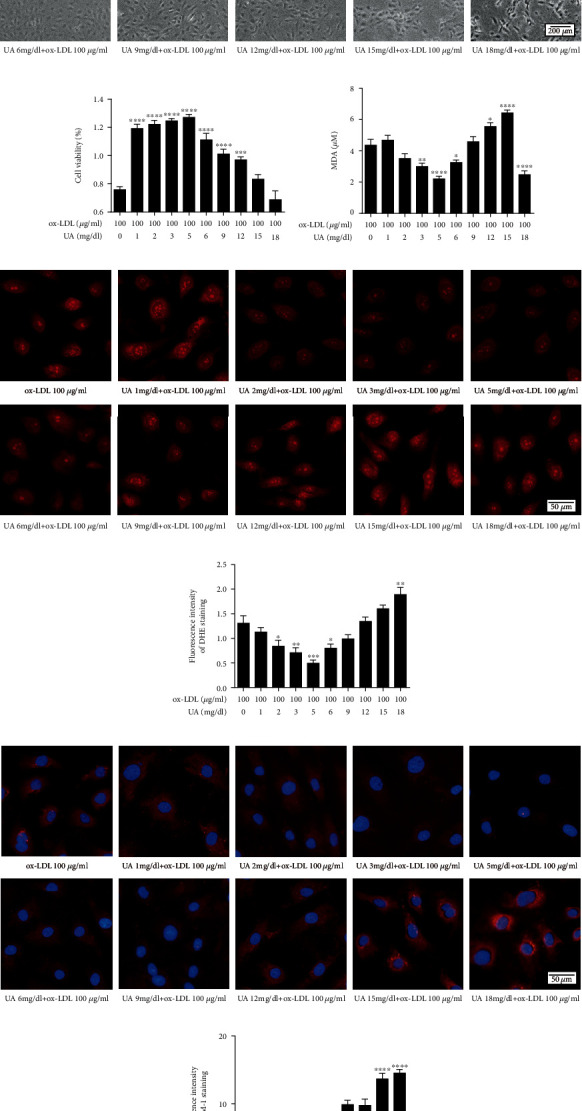
Changes of HUVECs under the stimulation of different concentrations of uric acid and fixed concentration ox-LDL. HUVECs were pretreated with 0, 1, 2, 3, 5, 6, 9, 12, 15, and 18 mg/dL uric acid 2 h before 100ug/mL ox-LDL stimulated. Changes in (a) cell morphology in different groups. Scale bar = 200 *μ*m. (b) Cell survival rate in different groups by CCK-8 assay. (c) MDA level in different groups. (d, e) DHE immunofluorescence image and densitometry analysis of immunofluorescent intensity of DHE. Scale bar = 50 *μ*m. (f, g) ICAM1 immunofluorescence image and densitometry analysis of immunofluorescent intensity of ICAM1. Scale bar = 50 *μ*m. DHE: dihydroethidium; HUVECs: human umbilical vein endothelial cells; ox-LDL: oxidized low-density lipoprotein; MDA: malondialdehyde. The statistical analyses were done using GraphPad Prism V-7.0 software. Data are expressed as the mean ± standard deviation (*n* = 3). One-way ANOVA was used in statistical analyses (*n* = 3/group). ^∗^*P* < 0.05, ^∗∗^*P* < 0.01, ^∗∗∗^*P* < 0.001, and ^∗∗∗∗^*P* < 0.0001 versus the control.

Treatment of HUVECs with UA and ox-LDL showed strongest cell viability at 5 mg/dL UA concentration compared with treatment with ox-LDL (100 *μ*g/mL) alone (Figures 3(a) and 3(b)). Pretreatment with 5 mg/dL uric acid significantly decreased MDA level by 53% (*P* < 0.05; Figure 3(c)). Similarly, the fluorescence intensity of DHE and ICAM1 were weakest after treatment with 5 mg/dL UA (Figures 3(d)–3(g)). These findings show the stable dose range for uric acid against HUVECs cell line for subsequent studies and show that the adverse effect of ox-LDL can be attenuated by uric acid. Subsequent experiments were performed using 5 mg/dL uric acid.

### 3.2. Uric Acid Attenuated HUVEC Injury Induced by ox-LDL

**Figure 4:**
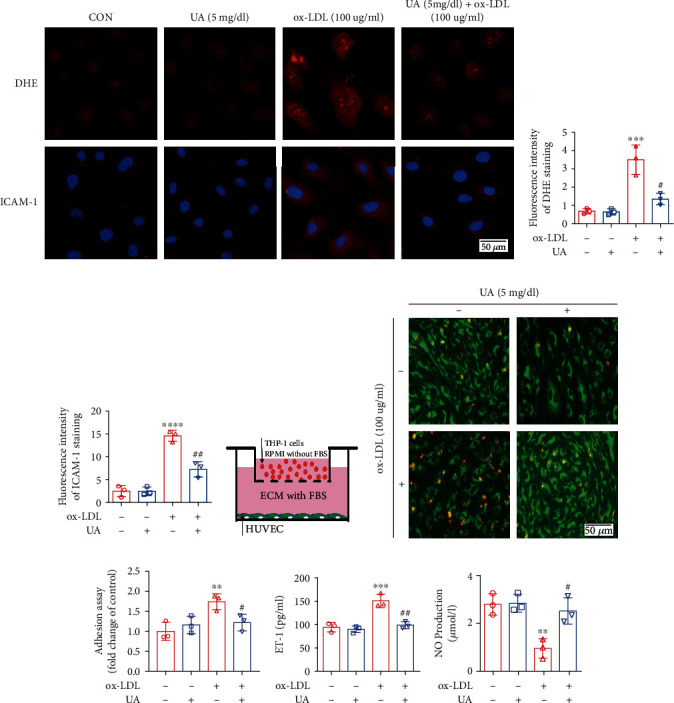
Appropriate concentration uric acid attenuated ox-LDL-induced injured in HUVECs. HUVECs were incubated with ox-LDL (100 *μ*g/mL) for 24 h, with or without uric acid (5 mg/dL) preincubated for 2 h. Or HUVECs were incubated with uric acid (5 mg/dL) alone for 24 h. (a) Representative immunofluorescence image of DHE and ICAM1. Scale bar = 50 *μ*m. (b) Densitometry analysis of immunofluorescent intensity of DHE in the HUVECs in different groups. (c) Densitometry analysis of immunofluorescent intensity of ICAM1 in the HUVECs in different groups. (d–f) Representative images showed adhesive monocytes to HUVECs under uric acid (5 mg/dL) treatment or ox-LDL (100ug/mL) treatment or uric acid (5 mg/dL)+ ox-LDL (100 *μ*g/mL). HUVECs were stained by Mito-Green, determined by green fluorescence, while THP-1 cells were stained by Dil, determined by yellow fluorescence. Quantitation of adhesive monocytes in different groups was presented in (f). (g) The effect of uric acid and ox-LDL on ET-1 expression in HUVECs. ET-1 released into the supernatant was measured by ELISA. (h) The effect of uric acid and ox-LDL on NO production in HUVECs. The results were independently repeated at least three times. DHE: dihydroethidium; HUVECs: human umbilical vein endothelial cells. The statistical analyses were done using GraphPad Prism V-7.0 software. Data are expressed as the mean ± standard deviation (*n* = 3). One-way ANOVA and unpaired *t*-test was used in statistical analyses (*n* = 3/group). ^∗^*P* < 0.05, ^∗∗^*P* < 0.01, ^∗∗∗^*P* < 0.001, and ^∗∗∗∗^*P* < 0.0001 versus the control. ^#^*P* < 0.05, ^##^*P* < 0.01, ^###^*P* < 0.001, ^####^*P* < 0.0001 versus the ox-LDL group.

Further, the effect of uric acid protected HUVECs against ox-LDL-induced inflammation and oxidative stress. Fluorescence intensity of DHE staining and ICAM-1 staining in the ox-LDL group increased by 386% and 484%, respectively (*P* < 0.05 vs. the control group; Figures 4(a)–4(c)). Notably, uric acid (5 mg/dL)+ox-LDL (100 *μ*g/mL) treatment reduced fluorescence intensity of DHE staining and ICAM-1 staining by 61.6% and 50.2%, respectively (*P* < 0.05 vs. the ox-LDL group; Figures 4(a)–4(c)). The control group and uric acid group showed no significant difference in fluorescence staining (*P* > 0.05). Furthermore, treatment with UA (5 mg/dL) significantly reduced ox-LDL-induced inflammatory responses in HUVECs, as shown by a significant decrease in monocyte adhesive capacity (*P* < 0.05 vs. the ox-LDL group; Figures 4(d)–4(f)). Levels of NO and ET-1 in the culture medium were determined to explore the effect of UA treatment on endothelial function in ox-LDL-induced HUVECs damage. ET-1 levels were slightly higher whereas NO production levels were decreased in the ox-LDL group (*P* < 0.05 vs. the control group; Figures 4(g) and 4(h)). In addition, the uric acid (5 mg/dL)+ox-LDL (100 *μ*g/mL) group showed significantly lower ET-1 levels and higher NO production levels compared with the levels in the ox-LDL group (*P* < 0.05; Figures 4(g) and 4(h)).

### 3.3. Activation of Nrf2 Is Consistent with the Protective Effect of Uric Acid against ox-LDL-Induced HUVEC Injury

**Figure 5:**
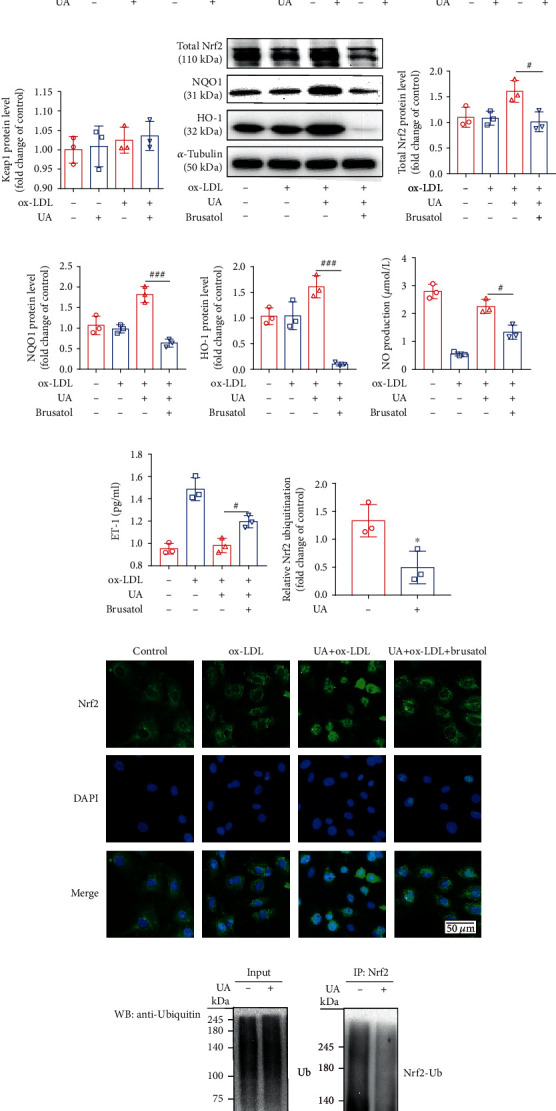
Nrf2 activation is associated with protective effect of uric acid-mediated on ox-LDL induced HUVECs injury. HUVECs were incubated with ox-LDL (100 *μ*g/mL) for 24 h, with or without uric acid (5 mg/dL) preincubated for 2 h. HUVECs were preincubated with brusatol (300 nM) for 2 h before incubated with UA (5 mg/dL)+ox-LDL (100 *μ*g/mL) for 24 h. (a–d) The cytosolic and nuclear compartments of HUVEC cells were fractionated, cell lysates were analyzed by Western blotting with primary antibodies against Nrf2 and Keap1. Protein levels were quantified by densitometry. *α*-Tubulin and histone H3 were used as internal controls. (e–h) Cell lysates were analyzed by Western blotting with primary antibodies against Nrf2, NQO1, and HO-1. Protein levels were quantified by densitometry. *α*-Tubulin was used as internal controls. (i) The effect of brusatol, uric acid, and ox-LDL on ET-1 expression in HUVECs. ET-1 released into the supernatant was measured by ELISA. (j) The effect of brusatol, uric acid, and ox-LDL on NO production in HUVECs. (k–m) Uric acid inhibited Nrf2 ubiquitination. Cells were treated with or without uric acid (5 mg/dL) for 6 h in the presence of MG132 (25 mM). For detecting ubiquitinated Nrf2, samples were subjected to IP with anti-Nrf2, followed by IB with an anti-Ub antibody. Nrf2 ubiquitination protein levels were quantified by densitometry. (l) Representative pictures showing the subcellular distribution of Nrf2 (FITC/green) in HUVECs. Nuclei were stained with DAPI (blue). Scale bar = 50 *μ*m. The statistical analyses were done using GraphPad Prism V-7.0 software. Data are expressed as the mean ± standard deviation (*n* = 3). One-way ANOVA was used in statistical analyses (*n* = 3/group). ^∗^*P* < 0.05, ^∗∗^*P* < 0.01, ^∗∗∗^*P* < 0.001, and ^∗∗∗∗^*P* < 0.0001 versus the control. ^#^*P* < 0.05, ^##^*P* < 0.01, ^###^*P* < 0.001, and ^####^*P* < 0.0001 versus the ox-LDL+UA group.

To explore the correlation between the protective effect of uric acid on ox-LDL-induced HUVEC injury and Nrf2 activation, cytosolic and nuclear compartments of HUVEC cells were fractionated and immunoblotted. Treatment with uric acid (5 mg/dL)+ox-LDL (100 *μ*g/mL) resulted in 2.33-fold and 3.44-fold increase in cytoplasmic Nrf2 protein levels and resulted in 1.62-fold and 4.14-fold increase in nuclear Nrf2 protein levels compared with the levels in the control and ox-LDL groups (*P* < 0.05; Figures 5(a)–5(c)). Additionally, determination of protein level of keap1 showed that uric acid and ox-LDL did not affect the expression of Keap1 (Figure 5(d)). To further verify that uric acid protects HUVECs injured by ox-LDL through the Nrf2 pathway, cells were cotreated with brusatol. Expression levels of total Nrf2, HO-1, and NQO1 were determined through Western blotting (Figure 5(e)). On the contrary to treatment with ox-LDL alone, uric acid pretreatment increased expression levels of total Nrf2, HO-1, and NQO1 by 60%, 63.5%, and 106.5%, respectively (*P* < 0.05; Figures 5(f)–5(h)). Also, ET-1 levels were significantly reduced compared with the levels in the ox-LDL group whereas levels of NO production were significantly increased in the UA+ox-LDL group (*P* < 0.05, Figures 5(i) and 5(j)). However, these changes were reversed by administration with brusatol. Further, immunofluorescence staining was performed to explore the subcellular distribution of Nrf2. Analysis showed that expression levels of Nrf2 in the control group were significantly higher in the cytoplasm, whereas lower expression levels were observed in the nucleus. On the contrary in the UA+ox-LDL group, Nrf2 was mainly localized in the nucleus. Treatment with the inhibitor showed reduction in Nrf2 levels in the nucleus compared with the levels in the UA+ox-LDL group (Figure 5(l)).

### 3.4. Uric Acid Suppressed Nrf2 Ubiquitination and Degradation

Nrf2 is a main regulator of the transcription of several antioxidant genes that protect cells against oxidative stress. In this study, treatment with 5 mg/dL uric acid significantly increased Nrf2 protein levels in nucleus and cytoplasm. Therefore, ubiquitin protein level and its interaction with Nrf2 were determined through immunoprecipitation. Notably, addition of proteasome inhibitor MG132 showed significant decrease in levels of ubiquitin protein in Nrf2 immunoprecipitation from uric acid treated cells. However, the protein expression level of Nrf2 showed a significant increase (Figures 5(m) and 5(k)).

### 3.5. Effects of Uric Acid on Inflammation and Oxidative Stress Caused by ox-LDL in Microfluidic Devices

**Figure 6:**
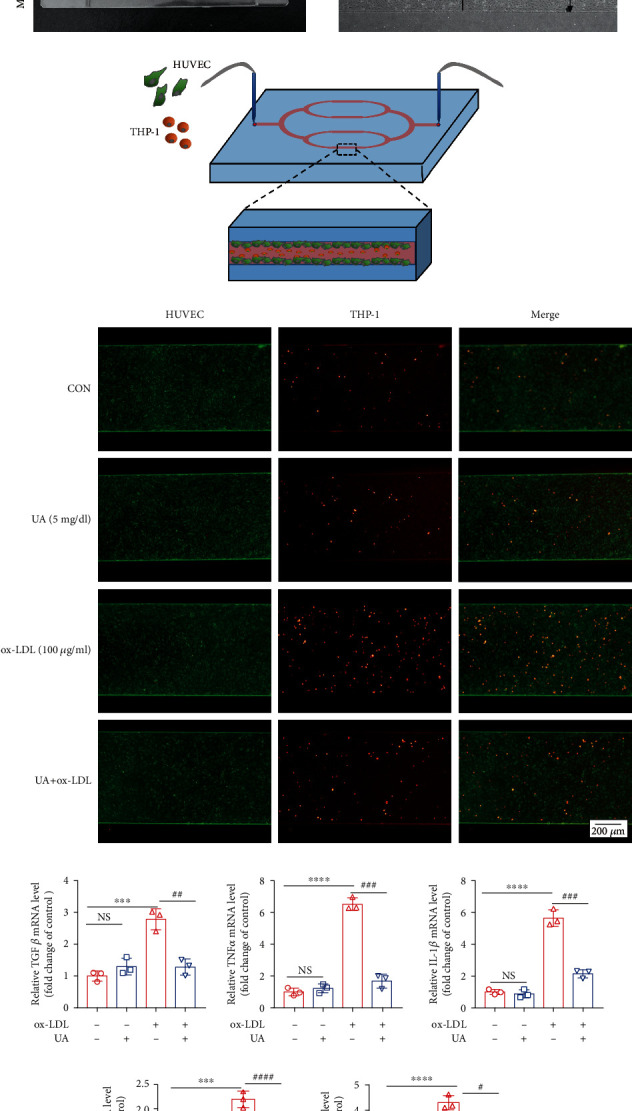
Effects of uric acid in microfluidic devices on inflammation and oxidative stress caused by ox-LDL in HUVECs. (a) Schematic diagram of vascular endothelial cells. (b) Photograph of a prototype. (c) A schematic diagram of cells in the micro chambers. (d) The microfluidic chambers were perfused with medium containing red THP-1 cells at velocity of 5 *μ*L/min. HUVECs were stained by Mito-Green, determined by green fluorescence. Scale bar = 200 *μ*m. (e) The mRNA levels of TGF*β*, TNF*α*, IL-1*β*, IL-6, and Nox4 were assessed by reserve transcription PCR. GAPDH served as loading controls. Group data were obtained by normalizing to GAPDH and expressed as fold of control values. The statistical analyses were done using GraphPad Prism V-7.0 software. Data are expressed as the mean ± standard deviation (*n* = 3). One-way ANOVA was used in statistical analyses (*n* = 3/group). ^∗^*P* < 0.05, ^∗∗^*P* < 0.01, ^∗∗∗^*P* < 0.001, and ^∗∗∗∗^*P* < 0.0001 versus the control. ^#^*P* < 0.05, ^##^*P* < 0.01, ^###^*P* < 0.001, and ^####^*P* < 0.0001 versus the ox-LDL group.

The vascular microfluidic model was used to verify the stable and appropriate concentration of uric acid for establishing a mouse model *in vivo* (Figures 6(a)–6(c)). HUVECs were incubated with ox-LDL (100 *μ*g/mL) with or without uric acid (5 mg/dL) for 24 h under low shear stress (5 *μ*L/min) in a microfluidic sdevice (Figure 6(b)). THP-1 cells and HUVECs were cocultured in a microfluidic system for 4 hours under low shear pressure (Figures 6(c) and 6(d)). Analysis showed that HUVECs treated with UA attenuated THP-1 cell adhesion compared with those treated with ox-LDL alone. qPCR analysis was used to determine mRNA expression level of TGF*β*, IL-1*β* Nox4, IL-6, and TNF *α* to further explore the anti-inflammatory and antioxidant effect of uric acid in ox-LDL-treated HUVECs. Treatment with ox-LDL significantly increased expression levels of above genes (*P* < 0.05 vs. the control; Figure 6(e)). Notably, pretreatment with uric acid (5 mg/dL) resulted in 53.9%, 59.6%, 93.8%, 32.3%, and 74.2% decrease in mRNA expression level of TGF*β*, IL-1*β*, Nox4, IL-6, and TNF*α*, respectively (*P* = 0.05 vs. the ox-LDL group; Figure 6(e)).

## 4. Discussion

**Figure 7:**
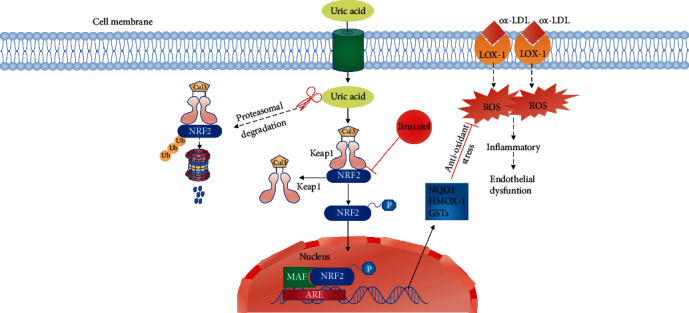
The potential protective mechanisms of UA on ox-LDL-induced HUVEC injury via the Keap1-Nrf2-Are pathway.

Uric acid is considered a neuroprotective agent for Parkinson's disease. And uric acid has direct and indirect neuroprotective effects [27, 28]. However, the mechanisms that underlie the protection of uric acid in cardiovascular remains poorly understood. In this study, we evaluated the effect of uric acid on ox-LDL-induced HUVECs damage. We found that ox-LDL (100 *μ*g/mL) reduced cell viability and increased the level of MDA, while the effect of uric acid (5 mg/dL) for 24 h reversed this effect. The findings of this study showed that UA was effectively inhibited ox-LDL-induced HUVEC damage in vitro. The protective effect was mediated through (1) inhibition of ROS production, (2) suppression of inflammation, and (3) inhibition of Nrf2 ubiquitination, induction of Nrf2 nuclear translocation, and induction of HO-1 and NQO1 gene expression as shown in Figure 7. Notably, protective effects caused by UA were reversed by treatment with brusatol. These findings showed that UA protected HUVECs from ox-LDL damage through induction of Keap1-Nrf2-ARE pathway activation.

Several essential mechanisms, including oxidative stress [29], vascular endothelial damage [30], and the release of inflammatory mediators [31], can initiate and exacerbate atherosclerosis, which is a contributing factor in many cardiac and cerebral vascular disorders. ox-LDL is implicated in the initiation and progression of atherosclerosis, through endothelial damage, adhesion molecule expression, and leukocyte recruitment and retention [32]. Accumulation of ox-LDL in the blood vessel wall can cause early vascular dysfunction, significantly decreasing NO production and increasing ROS [33]. These changes affect vascular endothelial function and promote atherosclerosis-related pathogenic processes. In previous studies, they demonstrated that ox-LDL (100-150 *μ*g/mL) exposure reduced cell viability [34, 35]. Consistent with the previous studies, the findings of the current study showed that high ox-LDL concentration (20-200 *μ*g/mL) induced cytotoxic effects, directly up-regulated production of ROS, significantly increased MDA level, and enhanced expression of ICAM1 in HUVECs (Figure 1). Therefore, we used ox-LDL as a drug to induce endothelial injury of HUVECs as an in vitro model to study the protective effect of uric acid on HUVECs.

Endothelial cells play a key role in maintaining the physiological functions of the cardiovascular system by regulating blood circulation, coagulation, angiogenesis, and inflammation [36]. The present study shows that uric acid can inhibit ROS production, and suppress inflammation responses of HUVECs exposed to ox-LDL (Figure 4), which is in accordance with a clinical trial study that reported using intravenous uric acid injections in healthy and diabetes volunteers can restore endothelial function in diabetes patients who are regular smokers [37]. Previous studies also reported that short-term administration can enhance the physiological effects of uric acid to avoid oxidative and free-radical driven tissue damage, such as sepsis. Early use of combination of uric acid and antioxidants results in a significant increase in cardiovascular hemodynamic [38]. Furthermore, administration of uric acid and vitamin C to healthy volunteers showed a significant enhancement in serum free-radical scavenging capacity from baseline, with no adverse effects observed after administration of 1,000 mg uric acid [39]. However, our results are also contradictory to previous reports on the effect of uric acid on cardiovascular disease. For example, epidemiological studies have also shown that serum uric acid levels are related to hypertension, dyslipidemias, diabetes, chronic kidney disease, atrial fibrillation, and cardiovascular events [5–11]. Basic experimental researches have shown that uric acid leads to endothelial dysfunction by activating NADPH oxidase, activating the RAAS system, and increasing oxidative stress and inflammation [24, 40]. In the current study, we observed that 0-5 mg/dL uric acid did not induce HUVEC damage (Figure 2), and 5 mg/dL uric acid can significantly reduce endothelial damage caused by ox-LDL (Figure 4). However uric acid (>5 mg/dL) had adverse effects on HUVEC (Figure 2) and >5 mg/dL uric acid had a synergistic effect on endothelial cell damage with ox-LDL (Figure 3). This phenomenon is because different concentrations of uric acid will produce different effects. As we all know, the physiological role of uric acid is a powerful antioxidant [12]. As the level of uric acid in the body increases, the absorption of uric acid into endothelial cells through uric acid transporters increases, leading to inflammation, oxidative stress, eNOS dephosphorylation, and endothelial dysfunction by reducing the bioavailability of NO [41].

This study showed that uric acid activated the Nrf2 antioxidant pathway and had a protective effect on endothelial damage induced by ox-LDL (Figure 5). Previous studies report that Nrf2 plays a key role in promoting cell redox homeostasis, thus maintaining cardiovascular health [42, 43]. Several experimental studies have determined the role of Nrf2 on expression of oxidative stress defense genes and protection of vascular health [43], and overexpression of Nrf2 in endothelial cells can reduce expression levels ofinterleukin1*β* (IL-1*β*), tumur necrosis factor (TNF), and protein 1 vascular cell adhesion (VCAM1) and protein 1 monocyte chemoattractant (MCP-1) [43]. However, low Nrf2 activity promotes to overexpression of proinflammatory chemokines and adhesion molecules in endothelial cells [44]. Our results showed that ox-LDL promoted oxidative stress, reduced Nrf2 protein expression, and Nrf2 nuclear translocation (Figure 5), which is inconsistent with previous reports [34, 45]. This contradiction may be because ox-LDL can induce endothelial cell senescence [46], and endothelial cell senescence is caused by transcriptional inhibition of Nrf2 expression [35, 47]. In previous studies, UA reduced the ubiquitination and degradation of Nrf2, promoted its nuclear translocation, and promoted the transcription and translation of antioxidant genes targeted by Nrf2, thereby providing neuroprotection to dopaminergic cells against 6-OHDA toxicity [48]. Therefore, the activation of Nrf2 may be an important mechanism of uric acid against atherosclerosis, but it has not been reported in the literature. In our study, UA stimulation increased the protein expression of Nrf2 in the nucleus and cytoplasm, suggesting that UA treatment can promote the translocation of Nrf2 into the nucleus and reduce Nrf2 ubiquitination (Figure 5). But it did not affect the expression of Keap1 protein, suggesting that uric acid can enhance the stability of Nrf2 at the protein level. It is generally believed that the chemical activation of Nrf2 is due to the separation of Nrf2 from Keap1, allowing Nrf2 to escape from Keap1-mediated proteasome degradation. This structure-activity relationship may be one of the mechanisms by which uric acid activates Nrf2. Structurally, UA assumes the form of a ketoenol tautomer, which can react with the cysteine residue of Keap1, so that the Nrf2 bound by Keap1 cannot be reached by ubiquitin ligase [49]. This mechanism is consistent with the recently reported 5,6-dihydrocyclopenta-1,2-dithio-3-thione (CPDT) and sulforaphane to activate Nrf2 and urate in 6-hydroxydopamine (6-OHDA) to activate Nrf2.

At the same time, consistent with previous studies [49, 50], we found that UA promoted the protein expression of HO-1 and NQO1 (Figure 5). HO-1 and NQO1 are regulated by Nrf2, which directly affects the body's antioxidant balance [20]. Importantly, after the preadministration of brusatol, the ability of UA to promote the protein expression of Nrf2/HO-1/NQO1 protein and nuclear translocation of Nrf2 was significantly hindered, suggesting that UA may regulate the expression of ARE-related genes by promoting the activation of Nrf2 and exerting an antioxidant effect. Brusatol treatment could inhibit the protective effect of UA on HUVECs damaged by ox-LDL. These data strongly proved the antioxidant effect and endothelial cell protection effect of UA activating the Nrf2 signaling pathway. However, this requires further research to evaluate this mechanism.

Currently, it is challenging to establish a stable uric acid concentration model in mice. Therefore, a microfluidic chip was used to further verify that an appropriate concentration of uric acid can reduce vascular damage caused by ox-LDL. The development of microfluidic organ models is a major field in bioanalytical chemistry, which is used in biological research, mainly in drug development. In addition to major organs including the lungs and liver, blood vessels are significant targets for biological examination [51]. Under static conditions, two dimensionally cultured experimental animals or cells are used to study blood vessels and related diseases. However, the results obtained from animal experiments are not always applicable to humans, and cells cultured *in vitro* are not a good model for vascular disease due to size differences and lack of blood flow. Three-dimensional (3D) primary cultures of human cells have been developed to imitate the human body in recent organ-on-chip studies. These *in vitro* models can be used to cultivate cells in extracellular (ECM) gels to imitate the organ microenvironment. In the microfluidic chip model, normal blood vessels, ox-LDL stimulated blood vessels, uric acid-stimulated blood vessels, and uric acid+ox-LDL costimulated blood vessels were simulated. Immunofluorescence results showed that ox-LDL significantly increased the expression of adhesion molecules on HUVECs, but 5 mg/dL uric acid reversed this phenomenon. The microfluidic chip also showed that uric acid suppressed inflammatory response and oxidative stress, as evidenced by decreased mRNA expression of TGF*β*, TNF*α*, IL-1b, IL-6, and Nox4 in HUVEC cells (Figure 6). Previous studies have demonstrated that HUVECs injured by ox-LDL secreted and expressed multiple proinflammatory cytokines, including IL-6, TNF*α*, IL-1*β*, and MCP-1 [34, 52], which is consistent with our *in vitro* results. These data indicated that the microfluidic model is feasible to mimic the in vivo environment. Further exploration is needed in the future.

## 5. Conclusions

Overall, the results showed that suitable concentration UA can attenuate oxidative stress and inflammatory response caused by ox-LDL in HUVECs through the Keap1-Nrf2-ARE pathway (Figure 7). The different concentrations of uric acid still have important guiding significance for clinical work. Therefore, although there is need to pay attention to hyperuricemia, the physiological effect of uric acid should be considered.

## Figures and Tables

**Table 1:** Primers used for quantitative real-time PCR analysis.

Gene	Forward Primer	Reverse primer
TGF*β*	5′-CGCCGAGCCCTGGACACCAACTA-3′	5′-GACAGCTGCTCCACCTTGGGCTT-3′
NOX4	5′- CCGAACACTCTTGGCTTACCTCC-3′	5′- AGCAGCCCTCCTGAAACATGCAA-3′
TNF*α*	5′- CACGCTCTTCTGCCTGCTGCACT-3′	5′- GGTACAGGCCCTCTGATGGCACCAC-3′
IL-1*β*	5′-TCCAGCTACGAATCTCCGACCAC-3′	5′-TGGGCAGACTCAAATTCCAGCTT-3′
IL-6	5′- AGCCACTCACCTCTTCAGAACGA-3′	5′- ACTTTTGTACTCATCTGCACAGCTC-3′
GAPDH	5′-CACCATCTTCCAGGAGCGAGATCCC-3′	5′-CCATCACGCCACAGTTTCCCGGAGG-3′

TGF*β*, transforming growth factor-*β*; Nox4, nicotinamide adenine dinucleotide phosphatase oxidase 4; IL-1*β*, Interleukin-1*β*; IL-6, Interleukin-6; TNF-*α*, Tumor necrosis factor; GAPDH, Glyceraldehyde 3-phosphate dehydrogenase.

## Data Availability

The corresponding author will provide the data used in this study upon request. Owing to privacy concerns, the data is not publicly accessible.
